# Joint line obliquity disruption negatively affects mid‐term patient‐reported outcomes following total knee arthroplasty

**DOI:** 10.1002/ksa.70389

**Published:** 2026-04-06

**Authors:** Patrick Pflüger, Alberto Pedrazzini, Lukas Fischer, Lukas Jud, Lazaros Vlachopoulos, Sandro F. Fucentese

**Affiliations:** ^1^ Department of Orthopedics Balgrist University Hospital University of Zurich Zurich Switzerland

**Keywords:** computed tomography, computer‐assisted surgery, knee osteoarthritis, patient‐reported outcome, total knee arthroplasty

## Abstract

**Purpose:**

The influence of change in coronal alignment on clinical outcomes following total knee arthroplasty (TKA) remains unclear. The Coronal Plane Alignment of the Knee (CPAK) classification can be broken up into arithmetic hip–knee–ankle (aHKA) angle and joint line obliquity (JLO) components, potentially identifying distinct failure mechanisms. This study investigated the influence of change in the magnitude and surgical accuracy of CPAK on patient‐reported mid‐term outcomes and the risk of aseptic revision.

**Methods:**

This retrospective cohort study included 216 patients (234 TKAs, median age 66 years, 65.3% women) undergoing patient‐specific instrumentation in TKA with a minimum follow‐up of 5 years. The change in CPAK was divided into aHKA and JLO components. Surgical accuracy was defined as deviation from the planned HKA angle. The primary outcome was the Western Ontario and McMaster Universities Osteoarthritis Index (WOMAC). A secondary outcome was aseptic revision of TKA. Multi‐stage method robust regression and logistic regression models were used, after adjustment for preoperative WOMAC scores and reintervention.

**Results:**

The median WOMAC score improved from 50 (interquartile range [IQR] 24.3) preoperatively to 11 (IQR 19) postoperatively. Our analysis of change in CPAK components revealed JLO disruption as the primary predictor associated with inferior outcomes, with each step change associated with worse WOMAC scores (*β* = 3.98, *p* = 0.02) and increased revision risk (odds ratio = 3.15, *p* = 0.04). In contrast, change in aHKA was not associated with either outcome.

**Conclusion:**

Within the CPAK framework, JLO disruption emerged as the primary predictor of patient‐reported inferior outcomes and risk of aseptic revision following TKA. Our current findings highlight the importance of preserving native JLO within personalized alignment strategies. Future prospective studies are needed to establish the acceptable degree of JLO change that maintains optimal joint kinematics and patient satisfaction.

**Level of Evidence:**

Level III.

**Abbreviations**
aHKA: arithmetic hip–knee–ankle angle
BMI: body mass index
CCI: Charlson ComorbidIty Index
CI: confidence interval
CPAK: Coronal Plane Alignment of the Knee
CT: computed tomography
DAIR: debridement, antibiotics and implant retention
FJS: Forgotten Joint Score
HKA: hip–knee–ankle angle
IQR: interquartile range
JLO: joint line obliquity
KA: kinematically aligned
LLR: long‐leg radiograph
MA: mechanically aligned
mLDFA: mechanical lateral distal femoral angle
mMPTA: mechanical medial proximal tibial angle
NWB: non‐weight‐bearing
OR: odds ratio
PROMs: patient‐reported outcome measures
PSI: patient‐specific instruments
TKA: total knee arthroplasty
WB: weight‐bearing
WOMAC: Western Ontario and McMaster Universities Osteoarthritis Index

## INTRODUCTION

Coronal Plane Alignment of the Knee (CPAK) is a pragmatic classification system based on arithmetic hip–knee–ankle angle (aHKA) and joint line obliquity (JLO) components [17]. By providing a practical framework with manageable phenotypic categories, CPAK has facilitated the comparison of outcomes across studies and enabled a common language for clinical research [1, 8]. Although CPAK has proven valuable for clinical research, its limitations as a two‐dimensional classification have been recognized [1]. With the evolution toward personalized knee arthroplasty, recent three‐dimensional analyses have identified additional rotational and multiplanar variations that may inform future phenotype‐based approaches [12].

Studies investigating the influence of CPAK phenotypic changes on patient‐reported outcome measures (PROMs) have yielded inconsistent results. Some studies have reported associations between JLO preservation and improved functional scores [19], while others found no significant relationships [4, 20]. A recent meta‐analysis demonstrated that JLO preservation was associated with improved short‐ to mid‐term outcomes, although substantial heterogeneity existed among the included studies [14]. This heterogeneity underscores the need for more nuanced analyses, as recent studies have demonstrated that outcomes vary based on the preoperative CPAK phenotype and degree of alignment modification [9, 10]. Additionally, existing studies have employed analytical approaches, including CPAK phenotypic changes [20], group comparisons [4], and modified JLO definitions [6], while limiting the duration of short‐term follow‐up from 1 [4, 9, 10, 20] to 2.5 years [19].

Given that two recent studies have demonstrated an association between postoperative JLO and the long‐term risk of aseptic revision [2, 22], longer‐term analyses evaluating functional outcomes and implant survival are warranted.

The goal of the present study was to investigate the influence of postoperative JLO disruption on patient‐reported mid‐term outcomes and the risk of aseptic revision following patient‐specific instrumentation in total knee arthroplasty (PSI‐TKA). We hypothesized that postoperative JLO disruption would be associated with inferior patient‐reported outcomes and an increased risk of aseptic revision.

## METHODS

### Included patients

This study was a retrospective review of a series of consecutively admitted patients who underwent primary PSI‐TKA (Medacta SA; MyKnee GMK©) between 2015 and 2020 at a single academic tertiary referral centre. A total of 332 PSI‐TKA procedures were identified. Patients with incomplete clinical or radiological data or documented refusal for inclusion were excluded. Of 280 patients assessed for eligibility, those contactable at least 5 years after index surgery were invited to complete a patient‐reported outcome questionnaire. Overall, 234 knees in 216 patients were included in the final analysis (Figure 1, Table 1). A comparison of baseline characteristics between included and excluded patients showed no significant differences in age (median value of 66 years for both groups), sex (65.3% vs. 71.9% women, respectively), body mass index (BMI) (30.4 vs. 32.0 kg/m^2^, respectively) or Charlson Comorbidity Index (CCI) (3 vs. 2, respectively), suggesting minimal selection bias.

**Figure 1:**
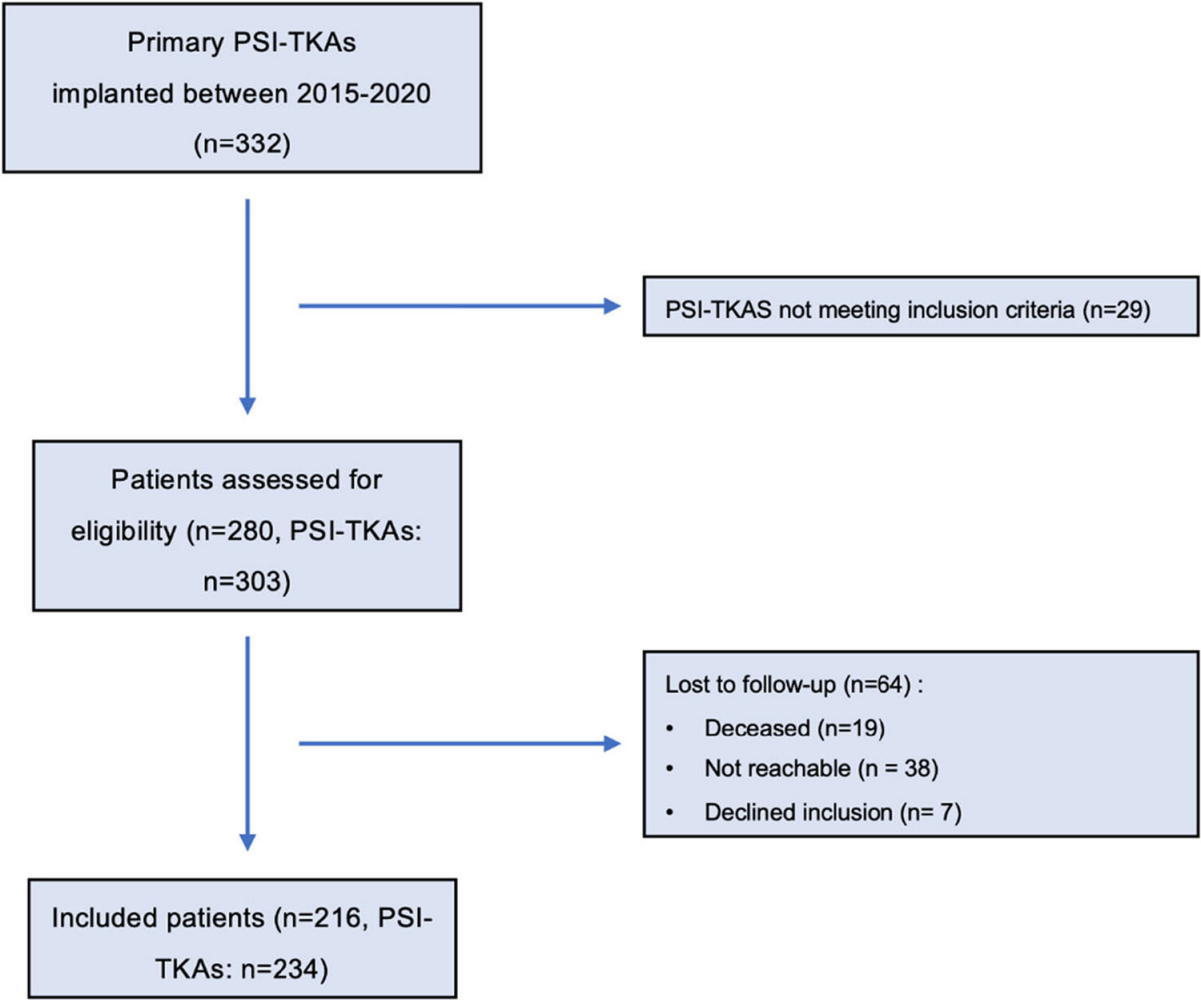
Flowchart presenting included and excluded patients. PSI‐TKA, patient‐specific instrumentation for total knee arthroplasty.

**Table 1:** Patient characteristics.

Demographic data	
Median age at surgery, years (IQR)	66 (14)
Women, *n* (%)	141 (65.3)
Bilateral, *n* (%)	18 (8.3)
Median BMI, kg/m^2^ (IQR)	30.4 (8.2)
CCI (IQR)	3 (2)

Abbreviations: BMI, body mass index; CCI, Charlson Comorbidity Index; IQR, interquartile range.

### Baseline information

All PSI‐TKA procedures were carried out in accordance with a standardized, previously described technique [18]. Femoral and tibial components were cemented, and a medial pivot insert was used in all cases. Patellar resurfacing was performed in only two knees at index surgery (0.9%). The alignment philosophy during the inclusion period aimed to achieve a mechanically aligned (MA) TKA (hip–knee–ankle [HKA] angle = 180°, femoral coronal angle = 90°, tibial coronal angle = 90°). Intraoperative adjustments to coronal alignment were made based on intraoperative stability testing when soft tissue balance could not be achieved with the planned alignment and were documented as the final targeted HKA. The targeted HKA was 180° in 94% of knees; in 3% of cases, the target was within 1° of neutral (*n* = 8), and in another 3% of patients, it was within 2° of neutral (*n* = 7).

Basic demographic data were collected, including age at the time of surgery, sex, BMI and CCI (Table 1).

### Outcome evaluation

All patients received regular follow‐ups at 12 weeks, 1 year and 2 years postoperatively. Standard radiographic assessment included anteroposterior knee radiographs, a preoperative non‐weight‐bearing (NWB) computed tomography (CT) of the lower extremity, and a weight‐bearing (WB) long‐leg radiograph (LLR). WB LLRs (EOS Imaging) obtained preoperatively and 1 year following PSI‐TKA were imported into mediCAD® software (2D V7.0; Hectec GmbH) to determine the following parameters: HKA, mechanical medial proximal tibial angle (mMPTA), and mechanical lateral distal femoral angle (mLDFA). Inter‐rater reliability (P. P. and A. P.) for HKA measurements was excellent (Intraclass correlation coefficient (ICC): preoperative ICC (ICCpreop): 0.99, 95% confidence interval (CI), 0.99–0.99, postoperative ICC (ICCpostop): 0.97, 95% CI, 0.96–0.98).

The CPAK classification types were determined preoperatively and 1 year postoperatively by calculating the aHKA (aHKA = mMPTA − mLDFA) and JLO (JLO = mMPTA + mLDFA). Knees were classified into nine phenotypes according to MacDessi et al. [17]. Surgical accuracy was measured as the absolute postoperative HKA deviation from the targeted HKA.

Preoperative knee function was routinely assessed by the patient using the Western Ontario and McMaster Universities Osteoarthritis Index (WOMAC) score. At least 5 years after TKA, patients were contacted to complete a follow‐up questionnaire. In cases of missing information, patients were contacted by telephone by three investigators (P. P., A. P. and L. F.) to collect the missing data.

Reinterventions following primary PSI‐TKA were analysed and categorized as follows: manipulation under anaesthesia (MUA), soft‐tissue debridement, debridement with antibiotics and implant retention (DAIR), secondary patellar resurfacing, open reduction and internal fixation (ORIF), TKA revision (exchange of at least one component and/or complete implant revision) and other procedures. Procedures due to periprosthetic infections were excluded in order to analyse the influence of coronal alignment on TKA revision.

### Primary and secondary outcomes

The primary outcome was the postoperative WOMAC score. The secondary outcome was the rate of aseptic revision of TKA.

This study was approved by the cantonal ethics committee of Zurich (BASEC No. 2025‐00602). Informed consent was obtained from all patients for both participation in and publication of the study. The study was conducted in accordance with the principles of the Declaration of Helsinki [21].

### Statistics

Study data were anonymously collected and managed using REDCap electronic data capture tools (Research Electronic Data Capture) [11]. RStudio software (R version 4.4.0 [2024‐04‐24]; PBC) was used for data processing, and a *p* value < 0.05 was considered statistically significant.

Continuous variables were expressed as median (interquartile range [IQR]) values and categorical variables as frequencies and percentages.

Constitutional alignment disruption was quantified using the CPAK classification, with changes categorized as 0 (unchanged), 1 (one‐step change) or ≥2 (two or more steps). CPAK change was divided into coronal (varus/neutral/valgus) and JLO (apex distal/neutral/proximal) components.

Postoperative WOMAC scores showed substantial positive skew (skewness = 1.48) and 12 influential outliers (Cook's distance > 4/*n*) were identified. Consequently, multi‐stage method (MM) robust regression was employed for the primary analysis, adjusted for preoperative WOMAC scores and reintervention excluding MUA. For aseptic TKA revision, multivariate logistic regression was used, including preoperative WOMAC scores, absolute postoperative HKA deviation and JLO change as predictors. Post hoc power analysis for the primary outcome (postoperative WOMAC score) demonstrated 99.3% power with the given sample size and five predictors. For the revision outcome (20 events, 3 predictors), the events‐per‐variable ratio was 6.7 (recommended ≥10), with an achieved power value of 80.8% to detect the observed effect (odds ratio [OR] = 3.15).

## RESULTS

The median age of included patients was 66 years (IQR 14), and 65.3% were women (*n* = 141) (Table 1). The median surgical accuracy was 2.1° (IQR 2.4), deviating from the targeted HKA (range: 0.0°–9.7°). Constitutional alignment was predominantly varus (Figure 2). The median follow‐up was 72 months (IQR 18).

**Figure 2:**
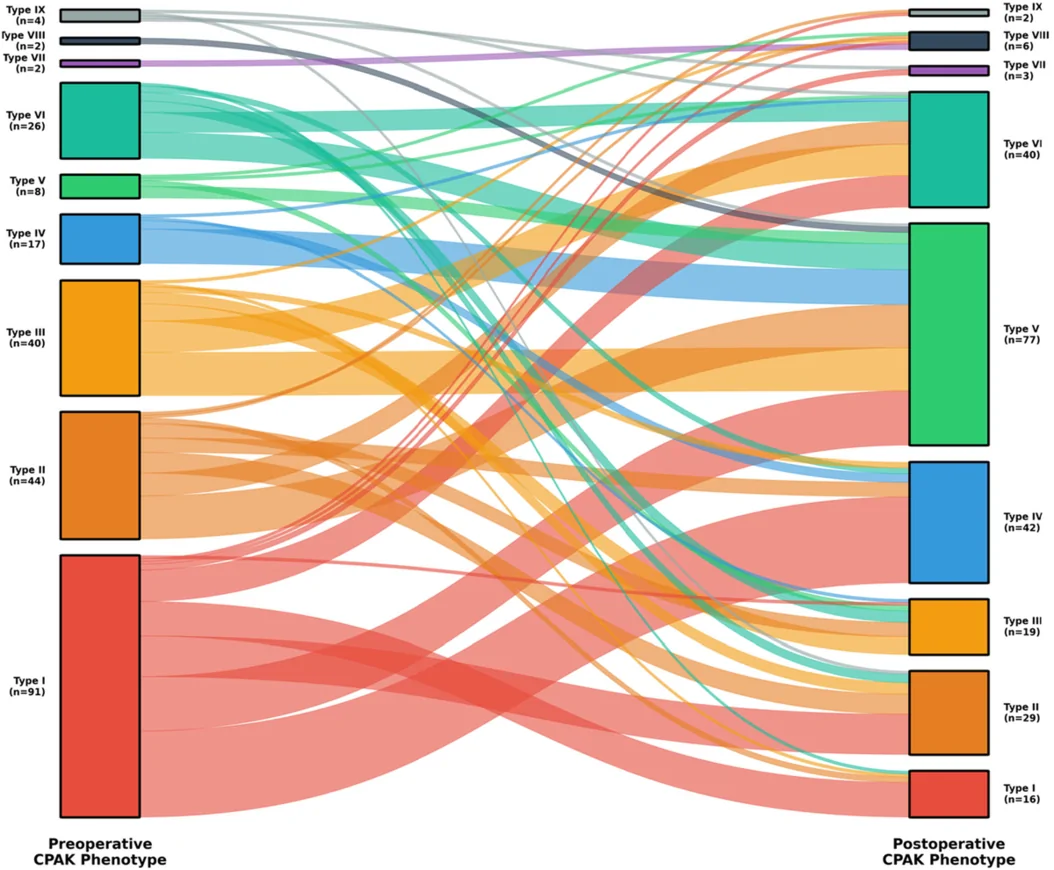
Pre‐ and postoperative CPAK phenotypes. CPAK, Coronal Plane Alignment of the Knee; *n*, number.

The change in CPAK and surgical accuracy had no significant correlation (*r* = 0.025, *p* = 0.71).

The majority of patients (87.6%, *n* = 205) reported that they would undergo the procedure again, with a median of 90% who fulfilled expectations (IQR 29).

Preoperative WOMAC scores had a median value of 50 (IQR 24), and postoperative WOMAC scores had a median of 11 (IQR 19). Thirty‐six TKAs (15.4%) underwent reintervention, of which nine (3.8%) had more than one reintervention (Table 2).

**Table 2:** Types of reinterventions.

Type of reintervention	
MUA, *n* (%)	8 (3.4)
Soft‐tissue debridement, *n* (%)	2 (0.9)
DAIR, *n* (%)	3 (1.3)
Secondary patellar resurfacing, *n* (%)	3 (1.3)
Aseptic TKA revision, *n* (%)	20 (8.5)

Abbreviations: DAIR, debridement, antibiotics and implant retention; MUA, manipulation under anaesthesia; TKA, total knee arthroplasty.

### Patient‐reported outcome

In multivariate MM robust regression, JLO change emerged as the primary predictor of patient‐reported outcomes (*β* = 3.98 per step change, 95% CI 0.78–7.18, *p* = 0.02), while aHKA change showed no association (*β* = 0.17; 95% CI −2.48 to 2.82, *p* = 0.89) (Table 3, Figure 3). CPAK change ≥2 showed a non‐significant trend towards worse postoperative WOMAC scores (*β* = 5.05, 95% CI −0.14 to 10.25, *p* = 0.06) (Figure 4). Median postoperative WOMAC scores increased progressively with JLO disruption magnitude (0 steps: 10 [IQR 15]; 1 step: 11 [IQR 19]; ≥2 steps: 23 [IQR 29]).

**Table 3:** Predictors of postoperative WOMAC score.

Variable	*β*, 95% CI	*p* value
CPAK change of 1	1.90 [−3.06, 6.86]	0.45
CPAK change ≥2	5.05 [−0.14, 10.25]	0.06
HKA deviation	0.63 [−0.33, 1.59]	0.19
Preoperative WOMAC	0.55 [−0.49, 1.6]	0.29
Reintervention (without MUA)	7.95 [2.44, 13.46]	<0.01

Abbreviations: CI, confidence interval; CPAK, Coronal Plane Alignment of the Knee; HKA, hip–knee–ankle angle; MUA, manipulation under anaesthesia; WOMAC, Western Ontario and McMaster Universities Osteoarthritis Index.

**Figure 3:**
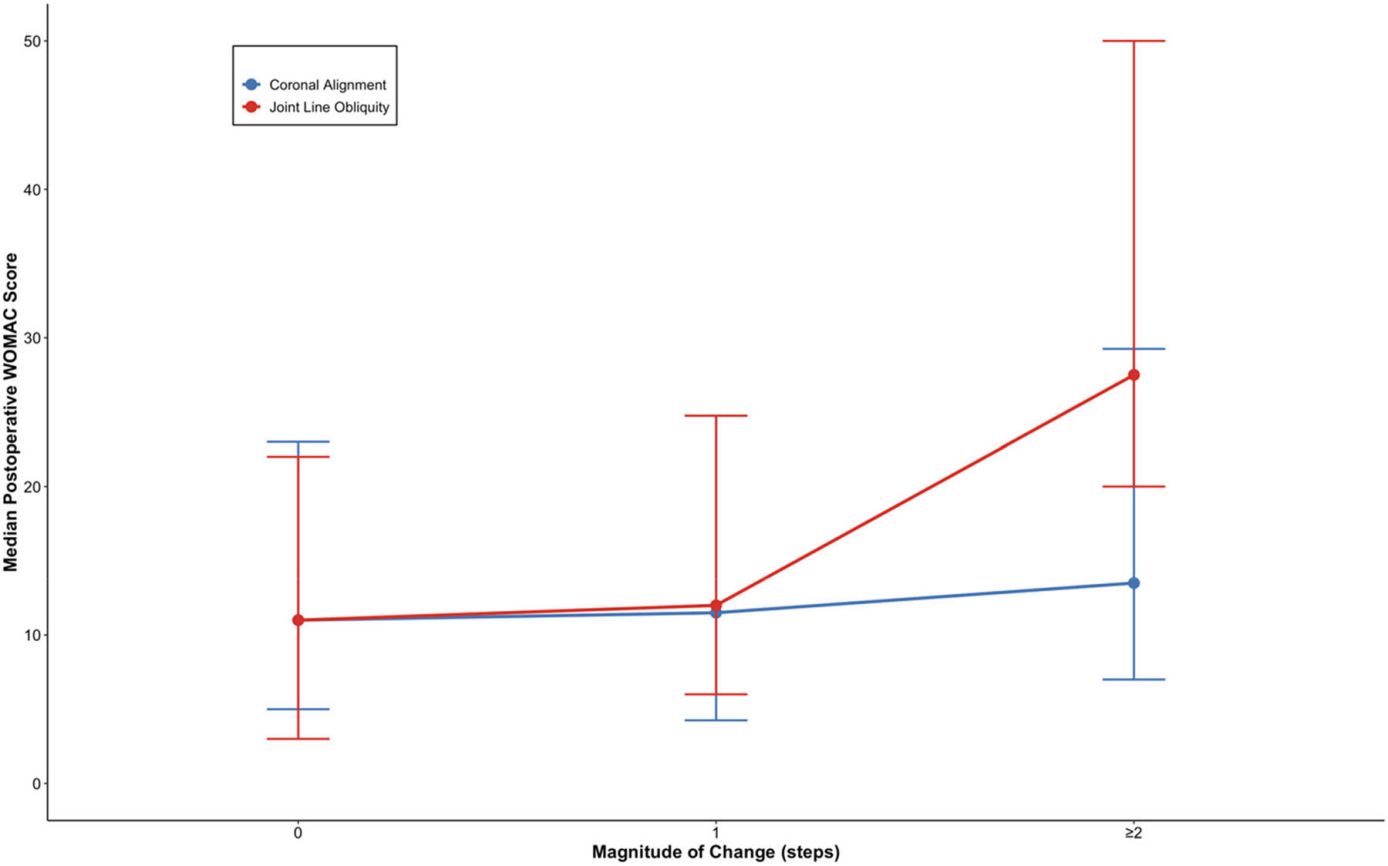
Postoperative WOMAC scores according to the magnitude of JLO change. JLO, joint line obliquity; WOMAC, Western Ontario and McMaster Universities Osteoarthritis Index.

**Figure 4:**
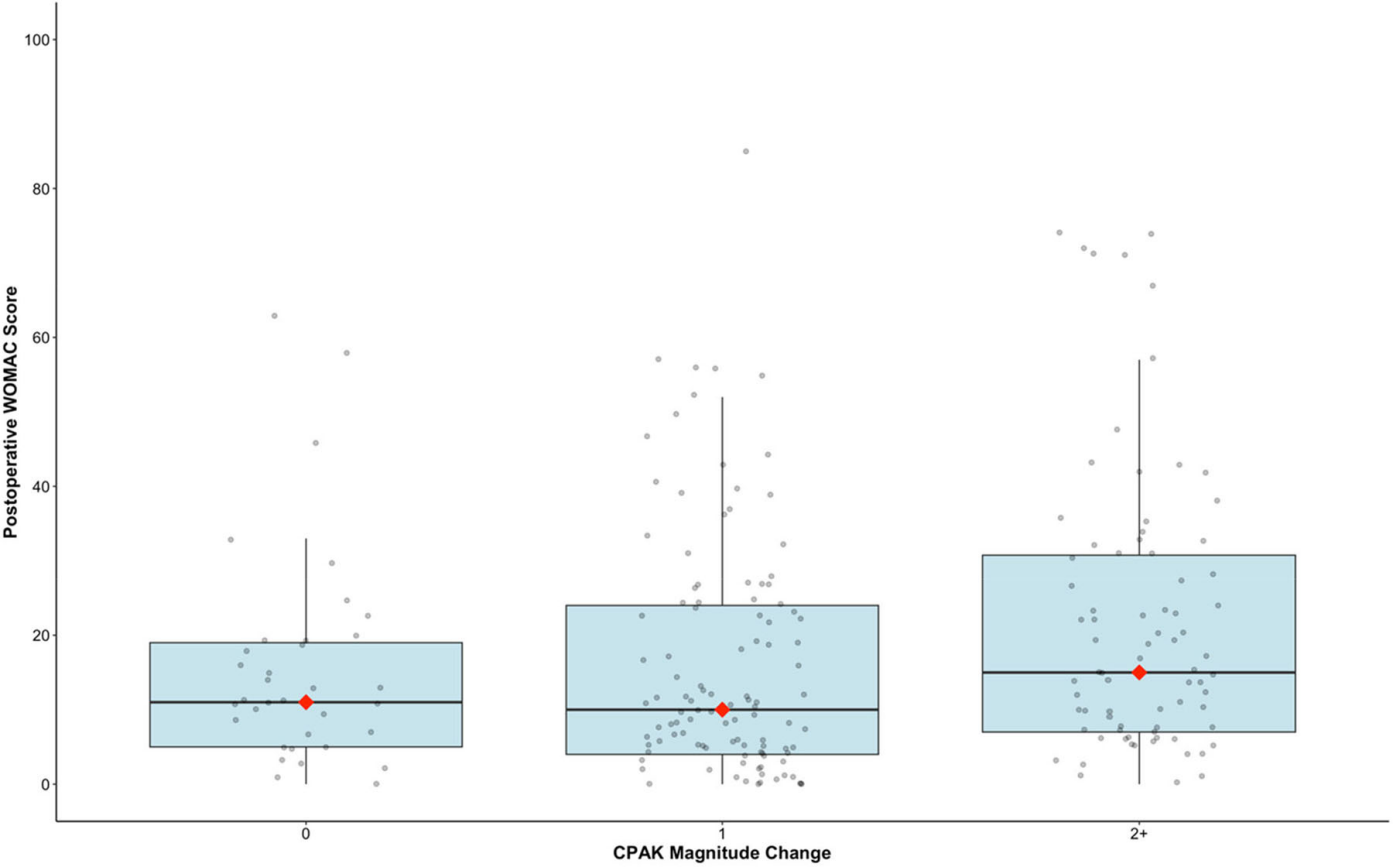
Postoperative WOMAC scores according to CPAK magnitude change. CPAK, Coronal Plane Alignment of the Knee; WOMAC, Western Ontario and McMaster Universities Osteoarthritis Index.

Surgical accuracy measured by HKA deviation was not associated with WOMAC score outcomes (Table 3). Reintervention excluding MUA was significantly associated with worse outcomes (*β* = 7.95, 95% CI 2.44–13.46, *p* < 0.01).

### Aseptic TKA revision

JLO change was the strongest alignment predictor of aseptic revision (odds ratio [OR] = 3.15 per step, 95% CI 1.09–10.41, *p* = 0.04), while HKA deviation showed a trend toward significance (Table 4). The preoperative WOMAC score was a significant predictor of revision (OR = 1.65 per 10‐point increase, 95% CI 1.15–2.38, *p* < 0.01). Notably, patients with a two‐step JLO disruption demonstrated a revision rate of 37.5% (3/8 patients), compared with 8.5% (8/94) for those with a preserved JLO (Figure 5). Coronal alignment change alone did not predict revision risk (OR = 1.51, *p* = 0.28).

**Table 4:** Predictors of aseptic revision of total knee arthroplasty.

Variable	OR, 95% CI	*p* Value
Preoperative WOMAC	1.65 [1.15, 2.38]	<0.01
HKA deviation	1.25 [0.96, 1.62]	0.10
JLO change	3.15 [1.09, 10.41]	0.04

Abbreviations: CI, confidence interval; HKA, hip–knee–ankle angle; JLO, joint line obliquity; OR, odds ratio; WOMAC, Western Ontario and McMaster Universities Osteoarthritis Index.

**Figure 5:**
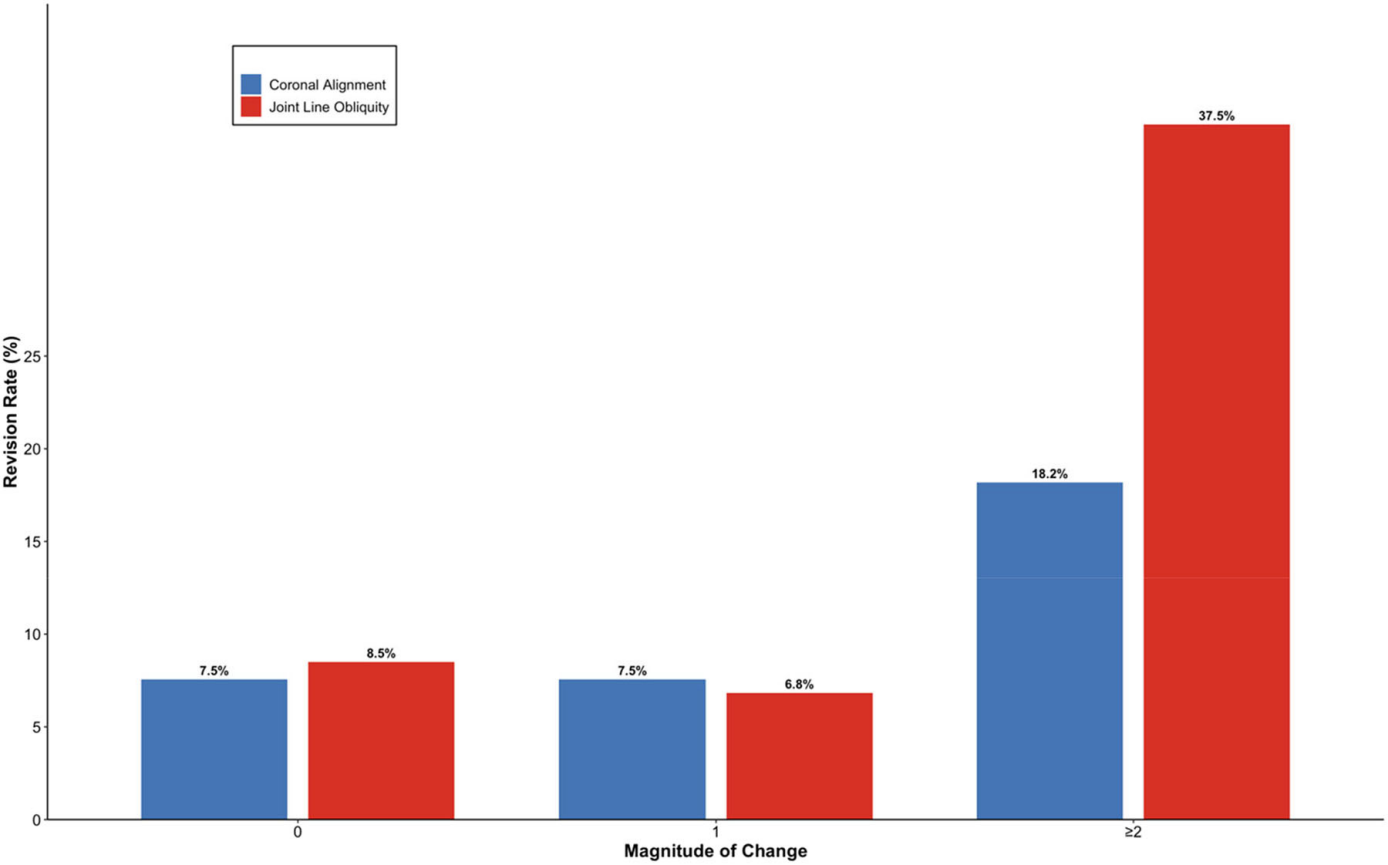
Aseptic TKA revision rates according to the magnitude of JLO change. CPAK, Coronal Plane Alignment of the Knee; JLO, joint line obliquity; TKA, total knee arthroplasty.

## DISCUSSION

The most important finding of our study was that JLO disruption was associated with patient‐reported inferior outcomes and increased risk of aseptic revision, thereby confirming our hypothesis. JLO change was significantly associated with WOMAC scores and revision risk, demonstrating a dose–response relationship with an increasing magnitude of disruption.

The influence of JLO on TKA outcomes has been examined in several recent studies with variable conclusions, largely attributable to methodological differences in how JLO is defined and assessed. A recent meta‐analysis by Jácome‐Pacheco et al., synthesizing 43 studies, found that joint line preservation in TKA was associated with improved outcomes. However, the analysis revealed substantial heterogeneity among the studies included in the meta‐analysis, with some reporting no significant association [14]. Notably, it was acknowledged that the primary role played by JLO may be limited without considering broader phenotypic alignment strategies. Importantly, JLO definitions varied considerably across the studies included in the meta‐analysis, contributing to substantial heterogeneity in the pooled results and limiting the ability to draw definitive conclusions about optimal JLO targets [14]. Tarassoli et al. analysed 296 TKAs with CT‐based measurements and found no correlation between alignment change and Knee Injury and Osteoarthritis Outcome Score‐based pain improvement at a short‐term follow‐up duration of 1 year [20]. Critically, their analysis employed absolute delta values, treating all deviations from native anatomy equally, regardless of direction—an approach that cannot detect the stepwise JLO disruption effect we have identified in our cohort. Bertugli et al. analysed 122 robotic arm‐assisted TKAs and found no significant differences in the Forgotten Joint Score‐12 (FJS‐12) values between patients with maintained versus changed CPAK phenotypes 1 year postoperatively [4]. However, this analysis was limited to short‐term follow‐up and employed subgroup comparisons between specific phenotype transitions (e.g., Types I–II, Types I–IV), thereby diminishing group sizes and statistical power. Similarly, applying a modified JLO definition (tibial joint line angle: TJLA), Calek et al. found no correlation between TJLA and FJS‐12 values 1 year postoperatively in 76 TKAs [6]. The Calek et al. study included two different TKA types and categorized patients into either horizontal or oblique TJLA groups, thereby further limiting statistical power given the small sample size [6].

In contrast to these negative findings, Sappey‐Marinier et al. examined a large cohort using the CPAK classification and found statistically reduced postoperative pain when apex distal JLO was restored [19]. However, their short‐term follow‐up of approximately 2.5 years may have been insufficient to detect clinically meaningful differences that typically emerge over longer observation periods. The influence of CPAK phenotypic modification on outcomes has been further demonstrated by two recent studies from Franceschetti et al. [9, 10]. In MA‐aligned TKAs, preoperative varus phenotypes showed inferior 1‐year PROMs compared with neutral and valgus phenotypes [9]. Subsequently, the same authors demonstrated that kinematic alignment (KA) yielded superior outcomes compared with mechanical alignment, specifically in CPAK I knees, while outcomes were comparable in CPAK IV knees [10].

Studies employing a modified JLO measurement (quantifying the tibial joint surface angle relative to the mechanical limb axis) have demonstrated associations between lateral JLO and inferior outcomes, with a focus on implant survivorship rather than patient‐reported outcomes. These studies employed various implant designs (posterior‐stabilized or cruciate‐retaining) and substantially longer follow‐up periods (>10 years), limiting direct comparisons with our current findings [2, 22]. Bae et al. classified a large cohort of TKAs into nine postoperative phenotypes and found that varus alignment combined with lateral JLO exhibited the worst long‐term survivorship after 15 years of follow‐up [2]. However, this study did not evaluate PROMs and therefore could not determine whether functional deficits preceded mechanical failure. Yang et al. employed a modified JLO classification and showed an association with aseptic revision after a mean follow‐up of >12 years with lateral JLO, demonstrating the highest failure rate [22]. Similar to other studies, patient‐reported outcomes were not assessed, leaving open the question of whether JLO affects patient function independently of its effect on implant longevity.

Preoperative patient factors are well‐established determinants of postoperative outcomes following TKA, justifying their inclusion as covariates in outcome analyses. Systematic reviews have consistently identified preoperative pain and PROM scores as among the strongest predictors of postoperative function and satisfaction [3]. Bourne et al. demonstrated that preoperative pain at rest and low baseline WOMAC scores independently predicted patient dissatisfaction [5]. Similarly, Kim et al. showed that patients with heightened preoperative pain sensitivity exhibited persistently inferior WOMAC scores through 1 year of follow‐up compared with those with lower sensitivity [15]. Consistent with this literature, preoperative WOMAC score emerged as a statistically significant predictor of aseptic TKA revision in our current analysis. Further, our data revealed that reintervention was associated with worse postoperative WOMAC scores, aligning with prior evidence that complications requiring additional interventions substantially increase the risk of patient dissatisfaction [5, 7].

Our study contributes to the evolving understanding of the influence of coronal alignment phenotypes on clinical outcomes. The observed 4‐point WOMAC score difference per JLO step change is below the established minimal clinically important difference thresholds (8–12 points). However, this effect accumulates with a greater magnitude of disruption, with two‐step changes producing approximately 8‐point differences that approach minimal clinically important difference thresholds. This dose–response relationship suggests that JLO should not be considered a binary variable (preserved vs. unpreserved) but as a measurable parameter reflecting the degree to which surgery has maintained or disrupted the constitutional knee phenotype of the patient. Our findings demonstrate that greater deviations from constitutional alignment result in progressively worse outcomes, raising the question of whether uniform MA remains appropriate for all patients. Hirschmann et al. recently demonstrated that only 15.4% of knees exhibit neutral alignment (within 1 standard deviation [SD] of the mean) across all coronal parameters, while the majority show normal constitutional variation (within 2 SD units) [13].

This study has several limitations that warrant consideration. The retrospective single‐centre design introduces the potential for selection bias and limits generalizability to other populations and surgical settings. The sample size provided adequate statistical power for the overall model but limited precision for marginally non‐significant effects, and the events‐per‐variable ratio of 6.7 for the revision analysis is below the recommended threshold, requiring conservative interpretation. The use of CPAK classification, while providing a practical framework for phenotypic assessment, has recognized limitations as a two‐dimensional coronal‐plane system [8]. This classification does not differentiate between the severity of coronal deformity or account for ligamentous laxity and fails to incorporate rotational and sagittal alignments [1]. Functional knee phenotyping (FKP) may offer an advantage in understanding the differences between similar coronal plane alignments [12]. Whether extended FKP may help to stratify patients further to improve TKA outcomes needs to be examined.

Further, CPAK matrix groups do not exhibit a direct correlation with specific extra‐articular deformity patterns [16], which may independently affect surgical planning and outcomes.

The use of mechanical alignment in our study cohort must also be recognized as a limitation, as recent studies have demonstrated that the effect of the alignment approach on PROMs varies according to the preoperative CPAK phenotype [9, 10]. Therefore, our findings may not be generalizable to kinematic or restricted kinematic alignment strategies. Future studies may investigate the midterm PROMs comparing MA with KA in light of the CPAK classification.

The relatively high reintervention rate may reflect referral‐centre bias and limit applicability to primary arthroplasty settings with lower complication rates. The use of a single‐implant design limits generalizability to other prosthesis types. Although we controlled for key confounders, unmeasured patient‐specific factors may contribute to the observed associations. These limitations affect the strength of our conclusions and highlight the need for prospective validation in multicenter cohorts.

## CONCLUSION

The division of CPAK magnitude change into its aHKA and JLO components showed JLO disruption to be the primary predictor of inferior patient‐reported outcomes and increased risk of aseptic revision at mid‐term follow‐up. Constitutional coronal alignment changes without concomitant JLO disruption were well‐tolerated. Our current findings highlight the importance of preserving native JLO within personalized alignment strategies. Future prospective studies are needed to establish an acceptable degree of JLO change that maintains optimal joint kinematics and patient satisfaction across various alignment strategies.

## AUTHOR CONTRIBUTIONS

Patrick Pflüger carried out the data curation, statistical analysis and drafted the manuscript. Alberto Pedrazzini and Lukas Fischer helped with data acquisition and processing. Lukas Jud, Lazaros Vlachopoulos and Sandro F. Fucentese participated in its design and helped draft the manuscript. All authors read and approved the final manuscript.

## CONFLICT OF INTEREST STATEMENT

Sandro F. Fucentese is a consultant for Medacta SA (Switzerland), Smith & Nephew (United Kingdom), Zimmer Biomet and Karl Storz SE & Co. KG (Germany). The remaining authors declare no conflict of interest.

## ETHICS STATEMENT

The study was conducted in accordance with the Declaration of Helsinki and approved by the local Ethics Committee (BASEC No. 2025‐00602). Written informed consent was obtained from all included patients.

## Data Availability

The data that support the findings of this study are available from the corresponding author upon reasonable request.
